# Resumption of Cochlear Implant Surgery under COVID-19 Pandemic Conditions

**DOI:** 10.3390/life11090929

**Published:** 2021-09-07

**Authors:** Henryk Skarzynski, Artur Lorens, Beata Dziendziel, Elzbieta Wlodarczyk, Anita Obrycka, Adam Walkowiak, Piotr Henryk Skarzynski

**Affiliations:** 1 World Hearing Center, Oto-Rhino-Laryngology Clinic, Institute of Physiology and Pathology of Hearing, 02-042 Warsaw, Poland; skarzynski.henryk@ifps.org.pl; 2 World Hearing Center, Implant and Auditory Perception Department, Institute of Physiology and Pathology of Hearing, 02-042 Warsaw, Poland; a.lorens@ifps.org.pl (A.L.); a.obrycka@ifps.org.pl (A.O.); a.walkowiak@ifps.org.pl (A.W.); 3 World Hearing Center, Rehabilitation Clinic, Institute of Physiology and Pathology of Hearing, 02-042 Warsaw, Poland; b.dziendziel@ifps.org.pl (B.D.); e.wlodarczyk@ifps.org.pl (E.W.); 4 World Hearing Center, Teleaudiology and Screening Department, Institute of Physiology and Pathology of Hearing, 02-042 Warsaw, Poland; 5 Heart Failure and Cardiac Rehabilitation Department, Faculty of Medicine, Medical University of Warsaw, 03-242 Warsaw, Poland; 6 Institute of Sensory Organs, 05-830 Kajetany, Poland

**Keywords:** COVID-19, cochlear implant, partial deafness treatment, adults, children

## Abstract

(1) Background: The novel coronavirus COVID-19 has been recognized by the World Health Organization as a public health emergency of international concern and has caused cancellation of elective cochlear implantation in many countries. This article sets out our experience with resuming cochlear implant (CI) surgery under COVID-19 conditions over a period of 3 months. In addition, early results of hearing preservation (HP) after CI surgery are presented; (2) Methods: We adopted epidemic management policies and procedures according to the National Consultant for Infectious Diseases recommendations. During preoperative visits, all patients were tested for COVID-19 with a RT-PCR test. One month postoperatively, HP values in the Partial Deafness Treatment (PDT) group of patients was established using the HEARRING group formula; (3) Results: Between January and March 2021, we performed 312 CI procedures in adult and pediatric patients. Of these, none were subsequently re-admitted to hospital and found to be COVID-19 positive. Postoperative audiometric results showed that complete or partial HP was achieved in more than half the PDT patients; (4) Conclusion: Cochlear implantation during the coronavirus disease pandemic is essential and, with careful planning, is perfectly feasible.

## 1. Introduction

The World Health Organization (WHO) launched its much-anticipated World Report on Hearing on the 3 March 2021 [1]. This Report follows on from WHO Resolution WHA70.13 [2], which urges member states to prioritize access to ear and hearing care (EHC) within national health plans and as part of universal health coverage. The rationale for this prioritization is the negative impact that unaddressed hearing loss can have on communication, speech, and language development, education, quality of life, employment, relationships, and cognition [3]. For the estimated 700 million people who require hearing intervention or rehabilitation, access to medical support, surgery, assistive devices, and high-quality hearing technology is vital. Access to technology remains a challenge, with only 10–23% of people globally using a hearing aid, despite potentially benefitting from one. The percentage for cochlear implants (CIs) is believed to be even lower. To address this challenge, the Report emphasizes that the newest hearing technologies, such as cochlear implants, are both efficacious and cost-effective. The Report therefore urges nations to take definite action, in particular by providing clear directions, identifying best practices, and providing cost-effective interventions for people with hearing loss. Given the importance and benefits of investing in a systematic scale-up of EHC services, the Report sets a global target that countries should aim to achieve: a minimum 20% increase in the effective coverage of EHC services from now to 2030 [1]. That means that by 2030 we should expect to see a 20% increase in the number of adults with hearing loss who use hearing technology such as auditory implants. 

However, the novel coronavirus COVID-19 (2019nCoV), which WHO has recognized as being a public health emergency of international concern (PHEIC) [4], has caused massive changes in EHC services delivery. After so-called lockdowns were introduced in many cochlear implant centers, CI surgeries were cancelled or conducted only to a very limited extent. National guidelines calling for the cancellation of all elective procedures present unique challenges to the health care system. Whereas there are some clear cases of elective interventions still being performed (such as benign cranial cosmetic defect) or emergency surgery (fracture, trauma, etc.), there is an unchartered middle ground in the case of CI interventions. Delaying a seemingly elective procedure can affect a deaf individual whose health is already vulnerable by further impacting their quality of life and their potential long-term health. Finally, extended lockdowns will drastically affect the chances of reaching the WHO target of a 20% increase in CI coverage. 

It follows that CI surgeries should resume, if appropriate permissions are given by the authorities and the CI center has adequate infrastructure in place in terms of equipment and manpower to conduct CI surgeries without compromising patient safety and care. Up to now there have been only two papers dealing with the issue of how to conduct CI surgeries during a pandemic [5,6]. The reported number of CI surgeries was only five in one center and 11 in the other. The authors discuss the changes that were necessary in preoperative, intraoperative, and postoperative protocols, and conclude that, from their limited experience, cochlear implantation during these difficult times can be undertaken if safety measures and guidelines to prevent infection are implemented at every level. 

In this manuscript, we report our experience with 312 CI surgeries done in the 3 months since resumption of our CI program. In addition, early results of hearing preservation after CI implantation are presented.

## 2. Materials and Methods

### 2.1. Participants

We resumed our CI program in January 2021, and between then and March, 312 CI procedures were performed at our tertiary referral center. Of the 312 patients, 188 (children, adolescents, and adults) were implanted unilaterally, with one of them (a child) undergoing a re-implantation. The remaining 124 patients underwent CI of the other ear (a sequential bilateral implantation procedure). The decision to qualify for a CI was made by a multi-disciplinary team (physicians, psychologists, teachers, speech therapists, and biomedical engineers). Depending on the age of the patient, the following preoperative audiological tests were performed.

- Auditory Brainstem Responses, ABR (for younger children)
- air-conduction hearing thresholds (for children older than 5 years and adults)
- a monosyllabic quiet word test (for patients with well-developed speech competencies who had indications that they were receiving insufficient benefits from conventional hearing aids—a result of 60% or less).

### 2.2. Safety and Quality of Hospitalization during COVID-19

The clinical staff were obliged to observe strict epidemiological rules set by the Institutional Committee for the Control of Hospital Infections for the duration of the pandemic. The clinical staff and healthcare professionals (i.e., hearing care professionals, nurses, physicians, psychologists, teachers, speech therapists, and biomedical engineers) used personal protective equipment (disposable gown, cap, gloves, surgical mask, goggles/face shield).

#### 2.2.1. Before Cochlear Implantation

All patients waiting to receive a CI were contacted by telephone 72 h before admission and asked about the state of their health and possible contact with COVID-19 infected persons. The patients received detailed information on the center’s epidemic management policies and procedures, which were in line with the recommendations of the National Consultant for Infectious Diseases. A minor, or a disabled patient, could be accompanied by only one person during the medical consultation.

For epidemiological purposes, five independent entrances to the center were established (incl. separate entrance for patients coming for hospitalization, outpatient visit or COVID-19 testing). A separate quarantine and isolation unit was also established. Before entering the building the patient passed through triage where their body temperature was measured. They were required to complete a health questionnaire, and received written information about the epidemiological rules (masks covering the mouth and nose, hand sterilizer, disposable gloves, social distancing, and following all instructions). After this, an entrance was designated. In many places around the center there are disinfectant stations for patients and information boards with instructions about how to reduce the risk of infection. During the visit, clinicians used (PPE)—disposable gown, cap, surgical mask, gloves, goggles/face shield. The consultation began with an epidemiological interview. For the needs of audiometric hearing tests, a special room was designated for preoperative patients. In the case of preoperative qualification for surgery, the patient was first referred for a COVID-19 test performed by the RT-PCR method (from the nasopharynx). Parents accompanying their children (when under 10 years of age) during the hospital stay were also subjected to PCR screening. Until the results of the COVID-19 test were received (normally within 12 h), the patient was hospitalized in a separate part of the facility (a patient isolation room) and remained under the care of dedicated staff. The patient could not leave the room until the test result was obtained. If the result was negative for COVID-19, the patient was then referred to the CI ward for hospitalization.

#### 2.2.2. CI Procedure

There are five operating theatres in our center, and each of them was sterilized before surgery. The interval between surgeries was at least 30 min. In the operating theatre, there was a minimum number of operating staff equipped with PPE (disposable gown, cap, surgical mask with HEPA filter, gloves, goggles) in accordance with the hospital’s guidelines. All CI procedures were conducted by the three senior surgeons from our tertiary referral center. In order to limit aerosol transmission and reduce the risk of infection, low-speed drills were used.

In our center we commonly use the minimally invasive surgical approach through the round window (following the Skarzynski 6-step procedure with full or partial insertion of the CI electrode as described by Skarżyński et al.) [7]. In the case of difficult or altered anatomical conditions, a cochleostomy was performed. Four different types of CIs were used: Med-El (GmbH, Innsbruck, Austria), Cochlear (Cochlear Ltd., Sydney, Australia), Advanced Bionics (USA, a division of Sonova), and Oticon Medical (Smørum, Denmark). The decision on which electrode to use was made based on preoperative air-conduction thresholds and inner ear anatomical conditions. 

After being extubated, patients were taken to an anaesthetic recovery room, where the staff also wore full PPE and all safety rules were respected. The patients were discharged within 3–4 days of admission. A 7-day home quarantine was recommended for each patient.

#### 2.2.3. After the CI Operation

Outpatient removal of the dressing and stitches and examination of the surgical site were scheduled 10 days after the surgery. A special office was set aside for these procedures. Each patient underwent an epidemiological procedure during triage, and the patient filled in another questionnaire on their state of health. Activation of the CI processor took place a month later. In order to maintain a sterile regime and limit the number of patients, additional days (over the weekend) were set aside exclusively for activation. Patients were informed about the option of tele-fitting and telerehabilitation via the use of the National Teleaudiology Network, which has been operating since 2009 [8,9].

### 2.3. Audiometric Assessment and Hearing Preservation

All patients (older children and adults) were given pre- and postoperative (1 month after implantation) audiometric assessment consisting of pure-tone audiometry in a soundproof cabin using calibrated audiometric earphones. Based on preoperative hearing thresholds and type of hearing loss, patients were divided according to Skarzynski’s concept of partial deafness treatment (PDT) [10,11,12]:

- Electric Stimulation (ES) in patients with non-functional hearing at all frequencies
- Electric-Acoustic Stimulation (EAS) in patients with mild-to-severe hearing loss at low frequencies and profound hearing loss at high frequencies
- Electric Complementation (EC) in patients with normal or slightly elevated thresholds at low frequencies and almost total deafness at higher frequencies
- Electro-Natural Stimulation (ENS) in patients with normal or slightly elevated thresholds at low and middle frequencies and severe-to-profound hearing loss at high frequencies.

In these groups, hearing preservation (HP) was calculated based on pure-tone audiometry at 11 audiometric frequencies (0.125, 0.25, 0.5, 0.75, 1, 1.5, 2, 3, 4, 6, and 8 kHz). HP is calculated using the HEARRING group formula [13]:

$$\mathrm{HP}=\left(1-\frac{PTApost-PTApre}{PTAmax-PTApre}\right)*100\;\left[\%\right]$$

Here, PTApre is the preoperative pure tone average, PTApost is the postoperative pure tone average, and PTAmax is the maximal testing level generated by a standard audiometer (assumed to be 110 dB HL). The results can be divided into: loss of hearing (no detectable hearing), minimal HP (range 1–25%), partial HP (range 26–75%), and complete HP (range > 75%).

## 3. Results

### 3.1. Safety and Quality of Hospitalization during COVID-19

Between January and March 2021, we made 371 telephone calls to patients waiting in the queue for CI surgery. The COVID-19 safety procedures were explained in detail. In the end, 31 patients (8%) declined to proceed with their implantation. During the preoperative medical consultation, another 13 patients were disqualified from surgery for reasons to do with anesthesia. The RT-PCR test for COVID-19 was performed on 471 people, both patients and adults accompanying a child. Some 2.8% of patients (adults) had a positive test result confirming COVID-19, and in 0.42% the test result was unclear. Finally, 312 patients qualified for implantation.

### 3.2. Participants

Characteristics of the study participants (age, gender, operated ear, etiology of hearing loss, unilateral or bilateral implantation, age at operation, and age at diagnosis of hearing loss) are presented in Table 1. They are divided into two groups: pediatric (i.e., children and adolescents) (*n* = 184) and adults (*n* = 128).

In the pediatric group, the majority of patients had pre- or perilingual hearing loss. Sudden deafness occurred in two children, in one at age 13 (etiology unknown) and in the other at age 2.5 years (after encephalitis). In the group of adult patients, nine had pre- or perilingual hearing loss. Progressive hearing loss was observed in 68 patients. Sudden deafness occurred in 51 patients, of which one patient lost hearing bilaterally during hospitalization due to a severe course of COVID-19. This patient previously had a bilateral high-frequency hearing impairment. This established that the episode of sudden sensorineural hearing loss and tinnitus (more burdensome on the right side) occurred during the fourth day of his hospitalization for COVID-19 and pneumonia. The patient did not require intubation and mechanical ventilation. Audiological tests revealed a profound hearing loss on the right side and moderate hearing loss on the left side. So far, several cases of hearing loss following COVID-19 have been reported in the literature [14,15,16,17].

In 269 patients (86.3%) a minimally invasive surgical approach through the round window was performed. In the remaining patients, due to abnormalities within the round window (invisible, atresia of the niche, hypoplastic), a cochleostomy was performed. Intraoperative bleeding occurred in nine patients (from the emissary vein, soft tissue, bone, mesotympanum lining, or cortex). During surgical access by the round window to the inner ear, oozing and leaks from the inner ear occurred in two patients. The reason for oozing in one patient was a congenital defect of the inner ear (incomplete cochlear partition type I); the etiology of the second patient was unknown, but there were no anatomical abnormalities of the inner ear. Significant intraoperative difficulties were experienced in one patient who lost hearing after head trauma with a fracture of the temporal bone. Intraoperatively, a damaged top of the cavity was found, which was filled with sharp bone fragments, and these were removed. The dura mater was exposed above the top of the cavity and there was leaking cerebrospinal fluid; this was blocked with the periosteum and tissue glue. Facial nerve dehiscence was found in three other cases.

### 3.3. Audiometric Assessment and Hearing Preservation

In 107 children and adolescents, due to their age or coexisting diseases (including cerebral palsy or mental handicap), an ABR test was performed before surgery. ABR results in 13 patients indicated asymmetric hearing loss (AHL: profound hearing loss in one ear and mild-to-moderate, moderate, or moderate-to-severe hearing loss in the other) or single-sided deafness (SSD: the second ear displays normal hearing). 

Audiometric assessment of hearing thresholds was possible in 205 patients. In this group, AHL or SSD was diagnosed in 93 patients. Based on preoperative hearing thresholds in the implanted ear, participants were divided into homogeneous groups according to Skarzynski’s concept of PDT: PDT-ENS in one patient, PDT-EC in five cases, PDT-EAS in 25 cases, and PDT-ES in 60 cases. In the remaining patients no residual hearing could be detected. 

**Figure 1:**
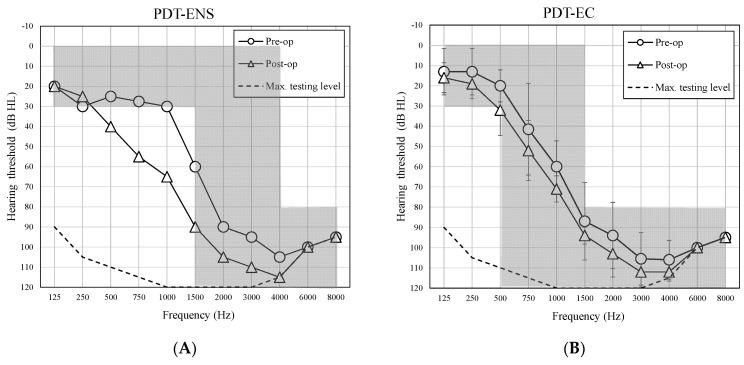

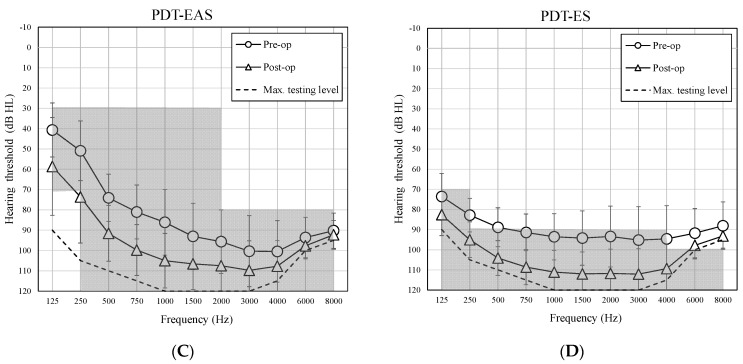
Average preoperative and 1-month postoperative air-conduction hearing thresholds in the implanted ear for groups of patients with PDT-ENS (**A**), PDT-EC (**B**), PTD-EAS (**C**), and PDT-ES (**D**). Error bars represent standard deviations.

The average pre- and postoperative air-conduction hearing thresholds in each group of PDT patients are shown in Figure 1. The HP outcomes at 1-month follow-up are presented separately for the PDT group in Table 2. Individual data including information about the type of implant, electrode, and pre-and postoperative audiometric results for PDT (ENS, EC, EAS, ES) patients can be found in the supplementary material Table S1.

Analyzing the results of HP, it is noted that the better the preoperative hearing thresholds, the better the results of HP after CI surgery. In the group of patients with PDT-ES, who had non-functional residual hearing, almost half achieved complete or partial HP after CI. In the group of patients with PDT-EAS, who had a good level of functional residual hearing, over 70% had complete or partial HP after surgery. There were four of five patients with PDT-EC who achieved complete HP (HP outcomes >80%). The HP result after CI for one PDT-EC patient was a partial HP of 73.9%. The one PDT-ENS patient had HP of 73.2%.

## 4. Discussion

The continuation of CI surgery during the COVID-19 pandemic can be deemed essential, although it depends on the availability of staff and implementing multiple safety precautions. Demonstrating the feasibility of CI surgery during the pandemic is of interest both nationally and internationally, especially given the unpredictable nature of the COVID-19 pandemic and the possibility of a resurgence of cases in the medium to long term. Where capacity allows, it is important to prioritize CI surgeries. However, several points need to be addressed.

First, there is the potential risk for transmission or development of COVID-19 during the peri-operative period, although the precise risk is still unknown. In our center, we performed 312 CI procedures during 3 months. Of these patients, none were subsequently re-admitted to hospital and found to be COVID-19 positive.

Second, the length of hospital stay is important and has long-term implications after the service is reintroduced. Alternative pathways, such as the introduction of telemedicine and teleconsultations, should be considered [18].

Third, during the COVID-19 period, there are likely to be additional problems with nonattendance based on patient anxiety and concerns about COVID-19 infection risk in the hospital. Our experience, however, was that by contacting patients using tele-consultancies to explain the safety procedures, we succeeded in achieving a non-attendance rate of 8%.

The first recommendations and guidance on the feasibility and safety of ENT care were published in 2020 [19,20]. Since then, various opinions and recommendations regarding the approach to CI implantation, especially in pediatric patients, have been published. Topskal et al. [21] suggested that, in order to maintain safety, CI implantation in prelingual profound sensorineural hearing loss children should be delayed by up to 12 weeks. The American Academy of Otolaryngology-Head and Neck Surgery recommended delaying CI surgeries in children by 3 to 6 months [22]. The British Cochlear Implant Group has recommended that CI in prelingual children with profound hearing loss take place within 3 months [5]. Likewise, the Cochlear Implant Group of India has recommended that paediatric CI implantation should not be made to wait for more than 3 months [6]. First experiences, recommended changes, and the feasibility of CI surgery during the COVID-19 pandemic have been reported by Mohammed [5] and Vaid [6]. Although these reports involve only small number of patients (five and 11 respectively), the authors suggest that CI procedures can still be done safely. 

Several major factors influenced our decision to resume our CI program. The first hopeful sign appeared with the launch of a national COVID-19 vaccination program for ‘group zero’, which includes both medical, administrative, and technical staff of centers involved in healthcare provision. Second, in our center the list of patients (children and adults) waiting in the queue for CI implantation before the pandemic was over 2000. In the case of adults, the average waiting time for the first CI surgery (non-urgent mode) was 3 years. It is still unknown how long the pandemic will last and how the disease curve will evolve. In Poland, the CI procedure and post-operative hearing rehabilitation program are financed under the universal health services, which is a contract awarded by the Polish National Health Fund. Stopping clinical activity is a potential failure to fulfill the health services contract, and this may lead to a contractual penalty and a reduction in the future budget of the CI program. As demonstrated by our results, CI procedures, while maintaining safety during the epidemic, are possible. However, this is associated with an increase in the cost of the procedure (such as PPE, changes in operating conditions, PCR screening tests, and increases in sterilization measures). On the other hand, it should be emphasized that during the pandemic, the Polish National Health Fund agreed to finance telerehabilitation services. Therefore, the number of tele-fitting and telerehabilitation services increased dramatically. These constituted 80% of the total rehabilitation services delivered at that time. Therapeutic strategies for treating deafness with a CI have been known for some years. Continuous technological improvement and minimally invasive surgery have led to a gradual widening in CI eligibility criteria [23]. The program of deafness treatment in Poland has been implemented since 1992. Since 2002, the CI program also covers patients with PDT [10]. In PDT patients, good hearing at low frequencies allows them to detect and discriminate all the vowels, but they are unable to discriminate most consonants, which are critical for speech intelligibility. Such a level of hearing does not allow patients to communicate effectively in everyday life, particularly in noisy backgrounds [24]. The purpose of a CI in this group is therefore to restore high frequency hearing, thereby improving speech discrimination and improving quality of life [25]. It can also prevent cognitive decline in older adults [26]. A CI is therefore in line with the newest WHO recommendation [1] for providing effective coverage of EHC services [24]. As demonstrated by the results of our observations, complete or partial HP was achieved in 100% of patients with PDT-EC or PDT-ENS, 72% with PDT-EAS, and 48% with PDT-ES. Promising long-term evidence regarding HP in subjects with residual low-frequency hearing has also been reported in recent studies by others authors [27,28,29]. Our results are very early and longer post-operative follow-up is needed, both to assess post-implantation HP and to improve speech discrimination with a CI.

At the time of writing, the expert team have published the latest recommendations for audiologists on safety and quality of service during the pandemic. The recommendations were developed during a discussion panel (organized virtually in June 2020) consisting of audiologists and surgeons who are members of the HEARRING group, a worldwide collaboration of more than 30 comprehensive hearing centers who deal with all aspects of hearing restoration with implantable devices [30]. The authors emphasize that the implementation of audiological service is vital, especially when deaf patients experience communication difficulties. 

## 5. Conclusions

CI implantation during the coronavirus disease pandemic is essential and, with careful planning, is perfectly feasible. Implementation of and adherence to strict safety measures enables a CI program to go ahead. Although the COVID-19 pandemic is still on-going, resumption of CI surgeries provides a positive stimulus for patients and their families. A CI provides both prelingual and post-lingual patients with a valuable chance to develop their auditory, speech, and language skills.

## Figures and Tables

**Table 1:** Characteristics of pediatric and adult patients.

Patient Characteristics	Pediatric	Adult
**Sex, No. (%)**		
**Male**	90 (48.9)	47 (36.7)
**Female**	94 (51.1)	81 (63.3)
**Implantation site, No. (%)**		
**Right**	73 (39.7)	67 (52.3)
**Left**	111 (60.3)	61 (47.7)
**Implantation procedure, No. (%)**		
**Unilateral**	69 (37.5)	119 (93.0)
**Bilateral**	115 (62.5)	9 (7.0)
**Etiology of hearing loss, No. (%)**		
**Acoustic trauma**	0	2 (1.6)
**Chronic otitis media**	0	12 (9.4)
**Congenital defect syndrome**	5 (2.7)	0
**Cytomegaly**	10 (5.4)	1 (0.8)
**COVID-19 virus**	0	1 (0.8)
**Genetic**	32 (17.4)	1 (0.8)
**Head trauma**	0	17 (13.3)
**Idiopathic SNHL**	95 (51.6)	64 (50.0)
**Inner ear malformation**	6 (3.3)	1 (0.8)
**Meniere’s disease**	0	5 (3.9)
**Meningitis**	0	5 (3.9)
**Mumps**	0	4 (3.1)
**Others**	2 (1.1)	5 (3.9)
**Otosclerosis**	0	6 (4.7)
**Ototoxic drug**	7 (3.8)	3 (2.3)
**Perinatal problems (i.a. prematurity, hypoxia)**	27 (14.7)	1 (0.8)
**Age at implantation in years, *M* (*SD*)**	6.9 (4.0)	53.6 (16.7)
**Duration of hearing loss in years, *M* (*SD*)**	6.6 (3.9)	16.1 (12.0)

Abbreviations: *M* = mean; *SD* = standard deviation.

**Table 2:** Hearing preservation outcomes at 1-month follow-up for groups of patients with PDT-ENS, PDT-EC, PDT-EAS, and PDT-ES.

	Hearing Preservation Evaluation			
	Complete HP *n* (%)	Partial HP *n* (%)	Minimal HP *n* (%)	Loss of Hearing *n* (%)
**PDT-ENS (*n* = 1)**	0 (0)	1 (100)	0 (0)	0 (0)
**PDT-EC (*n* = 5)**	4 (80.0)	1 (20.0)	0 (0)	0 (0)
**PDT-EAS (*n* = 25)**	7 (28.0)	11 (44.0)	5 (20.0)	2 (8.0)
**PDT-ES (*n* = 60)**	11 (18.3)	18 (30.0)	17 (28.3)	14 (28.3)

## Data Availability

The data that support the findings of this study are available from the corresponding author, upon reasonable request. The data are not publicly available due to legal restrictions (they contain information that could compromise the privacy of research participants).

## Supplementary Materials

The following are available online at https://www.mdpi.com/article/10.3390/life11090929/s1, Table S1: Information about the type of implant, electrode, and pre-and postoperative audiometric results for PDT (ENS, EC, EAS, ES) patients.
